# An Improved Ordinary State-Based Peridynamic Model for Granular Fractures in Cubic Crystals and the Effects of Crystal Orientations on Crack Propagation

**DOI:** 10.3390/ma17133196

**Published:** 2024-06-30

**Authors:** Yajing Gong, Yong Peng, Kui Wang, Song Yao, Shuguang Gong

**Affiliations:** 1 Key Laboratory of Traffic Safety on Track of Ministry of Education, School of Traffic & Transportation Engineering, Central South University, Changsha 410075, China; gongyj@csu.edu.cn (Y.G.); kui.wang@csu.edu.cn (K.W.); song_yao@csu.edu.cn (S.Y.); 2 School of Mechanical Engineering and Mechanics, Xiangtan University, Xiangtan 411105, China; gongsg@xtu.edu.cn

**Keywords:** peridynamics, cubic crystal, crystal orientation, granular fracture, crack propagation

## Abstract

Material anisotropy caused by crystal orientation is an essential factor affecting the mechanical and fracture properties of crystal materials. This paper proposes an improved ordinary state-based peridynamic (OSB-PD) model to study the effect of arbitrary crystal orientation on the granular fracture in cubic crystals. Based on the periodicity of the equivalent elastic modulus of a cubic crystal, a periodic function regarding the crystal orientation is introduced into peridynamic material parameters, and a complete derivation process and expressions of correction factors are given. In addition, the derived parameters do not require additional coordinate transformation, simplifying the simulation process. Through convergence analysis, a regulating strategy to obtain the converged and accurate results of crack propagation paths is proposed. The effects of crystal orientations on crack initiation and propagation paths of single-crystal materials with different notch shapes (square, equilateral triangle, semi-circle) and sizes were studied. The results show that variations in crystal orientation can change the bifurcation, the number, and the propagation path direction of cracks. Under biaxial tensile loading, single crystals with semi-circular notches have the slowest crack initiation time and average propagation speed in most cases and are more resistant to fracture. Finally, the effects of grain anisotropy on dynamic fractures in polycrystalline materials under different grain boundary coefficients were studied. The decrease in grain anisotropy degree can reduce the microcracks in intergranular fracture and the crack propagation speed in transgranular fracture, respectively.

## 1. Introduction

Cubic crystalline structures have been widely found in engineering materials. Their macroscopic mechanical and fracture characteristics are significantly influenced by microscopic properties such as grain orientation, size, and grain boundary strength [1]. For example, because of the symmetry of the cubic crystal, its equivalent elastic modulus changes periodically with the crystal orientation [2]. The crack propagation path of single-crystal silicon is significantly affected by the angle between the low-index crystal plane and the direction of the notch front [3,4].

Polycrystalline materials are composed of many grains with different orientations and grain boundaries between adjacent grains. The grain orientation distribution can elucidate the physical mechanisms of microstructure formation [5]. A grain boundary is a two-dimensional planar defect where various atomic reactions and processes are promoted or accelerated [6]. As a result, according to the difference in grain boundary strength, the main fracture modes observed in polycrystalline materials are intergranular fracture and transgranular fracture [7]. In general, as the grain boundaries become weaker and more misorientated, the transition from transgranular to intergranular fracture is likely to occur, i.e., intergranular-type cracks are more likely to occur at the grain boundaries where there is a large difference between the orientations of two grains [8]. Therefore, it is vital to study the effects of crystal orientation and grain boundary characteristics on the mechanical and fracture properties of crystal materials. 

The development of experimental techniques has led to a more in-depth understanding of the microstructure and properties of crystalline materials. However, in addition to the high cost caused by a large number of sample manufacturing and complex post-processing, many parameters during dynamic fracture are challenging to measure [9]. In order to provide a more effective method, many scholars have used different numerical methods to solve the fracture problems in crystalline materials. These include the cohesive zone model [10,11,12], extended finite element method [13,14,15], boundary element method [16,17,18], and phase field [1,19,20]. However, these methods are based on continuum mechanics theory and fundamentally have limitations in solving discontinuity problems [21]. 

Silling et al. [22] proposed a non-local theory, peridynamics (PD), which uses integral equations instead of partial differential equations in classical continuum mechanics (CCM) so it is not limited by material continuity and can spontaneously predict crack initiation and propagation. Recently, PD has been successfully applied to fracture analysis of polycrystalline materials. De Meo et al. [23] proposed a bond-state peridynamic (BB-PD) model for polycrystalline materials based on the cubic crystal. Similar to the definitions of matrix bonds and fibre bonds in the PD model of fibre-reinforced composite lamina [24], two types of bonds are defined in the direction of the relative position vector of a pair of material points in the BB-PD model for cubic crystals. One holds for all pairs of material points (Type-1), and the other is present only for the direction of some particular angles (Type-2), which is when the difference between the relative position vector direction of a pair of material points and the crystal orientation satisfies π/4 ± kπ/2 (k = 0,1,2,3). Subsequently, Li et al. [25] extended the application of the BB-PD model to the temperature field and studied the effects of grain size, grain boundary strength, and composite of materials. Lu et al. [21] investigated an application to the granular fracture behaviours of polycrystalline ice under dynamic loading conditions. However, the BB-PD model has limitations such as invariable Poisson’s ratios and does not support the constitutive model of plastic and incompressible materials [26]. Zhu et al. [27] developed an ordinary state-based peridynamics (OSB-PD) model to address the limitations of the BB-PD model and studied the effect of grain boundary strength, grain orientation, and grain size. Because of the arbitrariness of the orientation of polycrystalline materials, the direction of the relative position vector of all material point pairs easily mismatches the direction of the Type-2 bond in this type of model, making the model degenerate a model for isotropic materials. 

Anisotropy due to crystal orientation can be introduced into other ways in the PD model. Based on the OSB-PD model for isotropic materials, Li et al. [28] used a non-spherical influence function to characterise grain anisotropy, but this model requires additional calibration of the auxiliary parameters. Zhang et al. [29] added a periodic function with a period of π/2 to the PD strain energy density. Liu et al. [30] introduced a period of π/3 into the force density function to develop a chiral-dependent PD model for graphene sheets. In the BB-PD model, micro-modulus (or bond constant) is one of the critical parameters regarding the elastic properties of materials, the PD material parameters in the OSB-PD model have the same nature [26]. 

Therefore, this work aims to improve the OSB-PD model based on cubic crystals and introduce a periodic function containing crystal orientation into the PD material parameters. In addition, the material stiffness matrix after the coordinate transformation based on the crystal orientation is used, which allows the derived PD material parameters to contain the orientation angle, so no additional coordinate transformation is required during the simulation process.

The structure of this paper is arranged as follows. Section 2 outlines the fundamentals of OSB-PD theory for isotropic elastic materials. Section 3 establishes the improved OSB-PD theory for cubic crystals with arbitrary orientations and gives the derivations of PD material parameters, surface and volume correction factors, and the damage model. Section 4 uses the finite element method to verify the proposed PD model at first and then carries out the *m*-convergence and *δ*-convergence analysis on the static tensile and dynamic fracture problems. Section 5 presents the applications of the proposed PD model to the fracture behaviours of single crystals and polycrystalline materials, involving the effects of notch shape, size, and anisotropy degree. Section 6 summarises the main contributions of this work.

## 2. Fundamentals of the OSB-PD Theory

**Figure 1:**
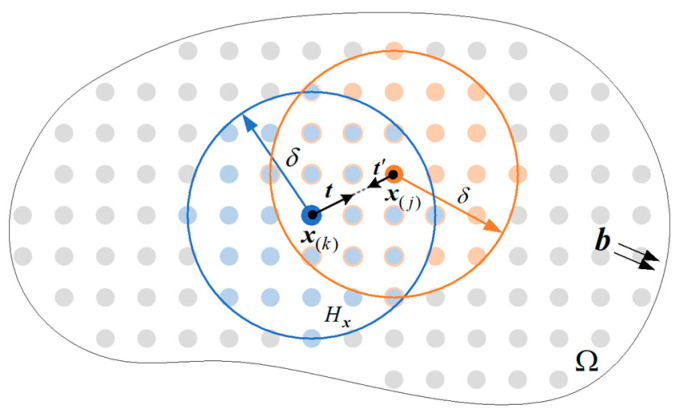
Non-local interaction between two material points in OSB-PD theory.

Peridynamics is a non-local theory of continuum mechanics, in which the mechanical responses of one material point $x_{\left(k\right)}$ in an object are related to other material points $x_{\left(j\right)}$ inside its neighbouring region $H_{x}$, called the horizon. In the two-dimensional plane problem, the horizon domain is a disk, and the horizon size is defined by a radius δ, as shown in Figure 1. The equation of motion at $x_{\left(k\right)}$ in the PD model is expressed by:

(1) $$\rho \ddot{u}(x_{\left(k\right)},t)=\int_{H_{x_{\left(k\right)}}}\left(t\left(u_{\left(j\right)}-u_{\left(k\right)},x_{\left(j\right)}-x_{\left(k\right)},t\right)-t(u_{\left(k\right)}-u_{\left(j\right)},x_{\left(k\right)}-x_{\left(j\right)},t)\right)dH_{x_{\left(k\right)}}+b(x_{\left(k\right)},t),$$

where b is the external force density; ρ is the material density; and u is the displacement vector. The connection between $x_{\left(k\right)}$ and $x_{\left(j\right)}$ is called the PD bond, so the vector $x_{\left(j\right)}-x_{\left(k\right)}$ is the bond vector. $t\left(u_{\left(j\right)}-u_{\left(k\right)},x_{\left(j\right)}-x_{\left(k\right)},t\right)$, or t, is the PD force density vector from $x_{\left(j\right)}$ to $x_{\left(k\right)}$ in the horizon domain of $x_{\left(k\right)}$, and $t(u_{\left(k\right)}-u_{\left(j\right)},x_{\left(k\right)}-x_{\left(j\right)},t)$, or $t^{\prime }$**,** is the one from $x_{\left(k\right)}$ to $x_{\left(j\right)}$ in the horizon domain of $x_{\left(j\right)}$.

In the OSB-PD model, the two pairwise force density vectors t and $t^{\prime }$ are parallel and opposite in direction but different in magnitude. Additionally, the force density vector is parallel to the deformed bond vector $y_{\left(j\right)}-y_{\left(k\right)}$, where $y_{\left(k\right)}$ and $y_{\left(j\right)}$ are the positions of $x_{\left(k\right)}$ and $x_{\left(j\right)}$ in the deformed configuration, and y=x+u. Thus, the two pairwise force density vectors can be calculated by

(2) $$t(u_{\left(j\right)}-u_{\left(k\right)},x_{\left(j\right)}-x_{\left(k\right)},t)=\frac{1}{2}A\frac{y_{\left(j\right)}-y_{\left(k\right)}}{\left|y_{\left(j\right)}-y_{\left(k\right)}\right|}$$

(3) $$t(u_{\left(k\right)}-u_{\left(j\right)},x_{\left(k\right)}-x_{\left(j\right)},t)=-\frac{1}{2}B\frac{y_{\left(j\right)}-y_{\left(k\right)}}{\left|y_{\left(j\right)}-y_{\left(k\right)}\right|}$$

where the symbol ‘|…|’ is the magnitude or norm of the vector,

(4) $$A=4ad\frac{\delta }{\left|x_{\left(j\right)}-x_{\left(k\right)}\right|}\Lambda_{\left(k)(j\right)}\theta_{\left(k\right)}+4b\delta s_{\left(k)(j\right)}$$

(5) $$B=4ad\frac{\delta }{\left|x_{\left(k\right)}-x_{\left(j\right)}\right|}\Lambda_{\left(j)(k\right)}\theta_{\left(j\right)}+4b\delta s_{\left(j)(k\right)}$$

where $\theta_{\left(k\right)}$ is the volume dilation. Normally, $\theta_{\left(k\right)}$ and $\theta_{\left(j\right)}$ are not equal.

(6) $$\theta_{\left(k\right)}=d\int_{H}\frac{\delta }{\left|x_{\left(j\right)}-x_{\left(k\right)}\right|}(\left|y_{\left(j\right)}-y_{\left(k\right)}\right|-\left|x_{\left(j\right)}-x_{\left(k\right)}\right|)dH$$

where $\Lambda_{\left(k)(j\right)}$ is the cosine of the angle between the relative position vectors of the initial configuration and the deformed configuration. $\Lambda_{\left(k)(j\right)}$ and $\Lambda_{\left(j)(k\right)}$ are equal in magnitude.

(7) $$\Lambda_{\left(k)(j\right)}=\frac{x_{\left(j\right)}-x_{\left(k\right)}}{\left|x_{\left(j\right)}-x_{\left(k\right)}\right|}\cdot \frac{y_{\left(j\right)}-y_{\left(k\right)}}{\left|y_{\left(j\right)}-y_{\left(k\right)}\right|}$$

where $s_{\left(k)(j\right)}$ is the stretch ratio, which is similar to the elastic strain in CCM theory.

(8) $$s_{\left(k)(j\right)}=\frac{\left|y_{\left(j\right)}-y_{\left(k\right)}\right|-\left|x_{\left(j\right)}-x_{\left(k\right)}\right|}{\left|x_{\left(j\right)}-x_{\left(k\right)}\right|}$$

For isotropic materials, if the temperature is neglected, the strain energy density at point $x_{\left(k\right)}$ is described by

(9) $$W_{\left(k\right)}=a\theta_{\left(k\right)}^{2}+b\int_{H}\omega_{\left(k)(j\right)}(\left|y_{\left(j\right)}-y_{\left(k\right)}\right|-\left|x_{\left(j\right)}-x_{\left(k\right)}\right|{)}^{2}dH$$

where $\omega_{\left(k)(j\right)}$ is the influence function

(10) $$\omega_{\left(k)(j\right)}=\frac{\delta }{\left|x_{\left(j\right)}-x_{\left(k\right)}\right|}$$

The variables a,b,and d are the PD material parameters to be solved, which can be obtained by comparing the strain energy densities under the simple loadings in PD to those in CCM. 

## 3. OSB-PD Theory for Cubic Crystals and PD Material 

### 3.1. Coordinate Transformation 

Because of the crystal orientation, the material stiffness matrix of the cubic crystals in the global coordinate system changes with the orientation angle. In CCM theory, the Voigt notations of the material stiffness matrix for cubic crystals are described below.

(11) $$C=\left[\begin{array}{cccccc} C_{11} & C_{12} & C_{12} & 0 & 0 & 0\\ C_{12} & C_{11} & C_{12} & 0 & 0 & 0\\ C_{12} & C_{12} & C_{11} & 0 & 0 & 0\\ 0 & 0 & 0 & C_{44} & 0 & 0\\ 0 & 0 & 0 & 0 & C_{44} & 0\\ 0 & 0 & 0 & 0 & 0 & C_{44} \end{array}\right]$$

In the assumption of plane stress, the material stiffness matrix C can be simplified by

(12) $$Q=\left[\begin{array}{ccc} Q_{11} & Q_{12} & 0\\ Q_{12} & Q_{11} & 0\\ 0 & 0 & Q_{44} \end{array}\right]$$

where $Q_{11}=\frac{C_{11}^{2}-C_{12}^{2}}{C_{11}}$, $Q_{12}=\frac{C_{11}C_{12}-C_{12}^{2}}{C_{11}},$ and $Q_{44}=C_{44}$.

**Figure 2:**
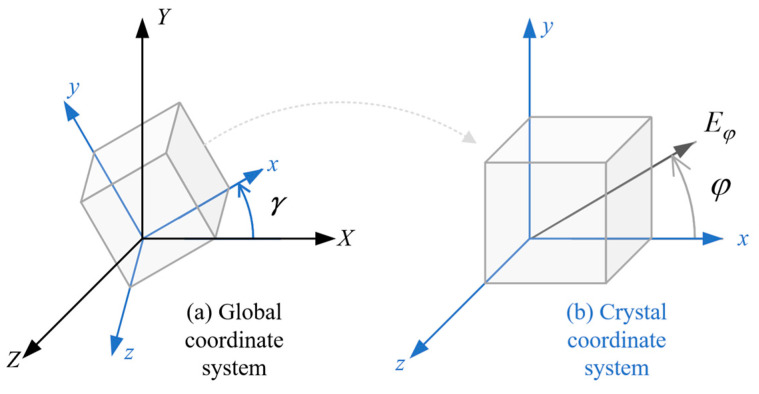
The relationship between (**a**) the global coordinate system and (**b**) the crystal coordinate system.

When considering a crystal orientation angle γ, there is a transformation between the crystal coordinate system and the global coordinate system, as shown in Equation (13) and Figure 2,

(13) $${\left[\begin{array}{c} X\\ Y \end{array}\right]}^{global}=\left[\begin{array}{cc} \cos\gamma & -\sin\gamma \\ \sin\gamma & \cos\gamma \end{array}\right]{\left[\begin{array}{c} x\\ y \end{array}\right]}^{crystal}$$

so that in the global coordinate system, the reduced material stiffness matrix with an orientation angle γ after the coordinate transformation is calculated as

(14) $$R=\left[\begin{array}{ccc} R_{11} & R_{12} & R_{14}\\ R_{12} & R_{11} & R_{24}\\ R_{14} & R_{24} & R_{44} \end{array}\right]=\left[\begin{array}{ccc} \frac{C_{11}D_{11}-C_{12}^{2}}{C_{11}} & \frac{C_{11}D_{12}-C_{12}^{2}}{C_{11}} & D_{14}\\ \frac{C_{11}D_{12}-C_{12}^{2}}{C_{11}} & \frac{C_{11}D_{11}-C_{12}^{2}}{C_{11}} & D_{24}\\ D_{14} & D_{24} & D_{44} \end{array}\right]$$

where

(15) $$\left\{\begin{array}{l} D_{11}=C_{11}-2\mu \sin^{2}\gamma \cos^{2}\gamma \\ D_{12}=C_{12}+2\mu \sin^{2}\gamma \cos^{2}\gamma \\ D_{14}=\mu (\sin^{3}\gamma \cos\gamma -\sin\gamma \cos^{3}\gamma )\\ D_{24}=\mu (\sin\gamma \cos^{3}\gamma -\sin^{3}\gamma \cos\gamma )\\ D_{44}=C_{44}+2\mu \sin^{2}\gamma \cos^{2}\gamma \\ \mu =C_{11}-C_{12}-2C_{44} \end{array}\right.$$

Similarly, in the condition of plane strain, the reduced matrix with a crystal orientation γ is

(16) $$T=\left[\begin{array}{ccc} T_{11} & T_{12} & T_{14}\\ T_{12} & T_{11} & T_{24}\\ T_{14} & T_{24} & T_{44} \end{array}\right]=\left[\begin{array}{ccc} D_{11} & D_{12} & D_{14}\\ D_{12} & D_{11} & D_{24}\\ D_{14} & D_{24} & D_{44} \end{array}\right]$$

The matrices R and T are used to derive the PD material parameters under the assumptions of plane stress and plane strain, respectively.

### 3.2. PD Material Parameters and Force Density Vectors 

In BB-PD, the micro-modulus is an intrinsic parameter of the material that represents the stiffness of the bond between a pair of material points and is directly related to elastic constants in CCM theory [31]. In the OSB-PD model for isotropic materials, *d* is independent of the material type. *a* and *b* are proportional to the elastic constants of materials, but *a* is relevant to the volume dilatation. Thus, *b* is the critical PD parameter of the associated intrinsic material properties, similar to the micro-modulus.

For cubic crystals, the elastic properties are different depending on the axial angle φ in the crystal coordinate system, as shown in Figure 2b. In the two-dimensional cases, the equivalent elastic modulus $E_{\varphi }$ at any direction of the crystal axis is calculated by

(17) $$1/E_{\varphi }=s_{11}+(2s_{12}-2s_{11}+s_{44})(\sin^{2}\varphi \cos^{2}\varphi )$$

where $s_{11}=\frac{C_{11}+C_{12}}{C_{11}^{2}+C_{11}C_{12}-2C_{12}^{2}}$, $s_{12}=\frac{-C_{12}}{C_{11}^{2}+C_{11}C_{12}-2C_{12}^{2}},$ and $s_{44}=\frac{1}{C_{44}}$ are the components of the compliance matrix of cubic crystals [2]. 

It is evident in Equation (17) that the equivalent elastic modulus for cubic crystals has a period of *π*/2. In order to make the PD material parameters match the corresponding anisotropic material parameters at an arbitrary crystal orientation and axial angle of the crystal, the idea is to correlate the PD material parameter *b* with the axial angle of the crystal φ. Assume that *b* depends on φ and has a period of *π*/2, by comparison with Equation (17), it is constructed by

(18) $$b=b_{1}+b_{2}\sin^{2}\varphi \cos^{2}\varphi$$

By substituting it into the equation of the strain energy density at $x_{\left(k\right)}$ (Equation (9)), the following is obtained

(19) $$W_{\left(k\right)}=a\theta_{\left(k\right)}^{2}+\left(b_{1}+b_{2}\sin^{2}\varphi \cos^{2}\varphi \right)\int_{H}\omega_{\left(k)(j\right)}(\left|y_{\left(j\right)}-y_{\left(k\right)}\right|-\left|x_{\left(j\right)}-x_{\left(k\right)}\right|{)}^{2}dH$$

Similar to isotropic materials, the PD material parameters $a,b_{1},b_{2},and\;d$ for cubic crystals can be solved by comparing the strain energy density under simple loading in PD and CCM theory. The derivation process is shown in Appendix A for the sake of brevity. Then, the solutions of the four PD material parameters are

(20) $$\left\{\begin{array}{l} a=\frac{1}{2}\left(R_{12}-R_{44}\right)\\ b_{1}=\frac{3}{2\pi h\delta^{4}k}\left[\left(10\pi k_{1}+3\pi k_{3}\right)(R_{11}-R_{12})-2\left(14\pi k_{1}+\pi k_{3}\right)R_{44}\right]\\ b_{2}=\frac{24\left[2R_{44}-(R_{11}-R_{12})\right]}{h\delta^{4}k}\\ d=\frac{2}{\pi h\delta^{3}} \end{array}\right.$$

where $k=\pi k_{3}-2\pi k_{1}$, $k_{1}=\sin^{2}\gamma \cos^{2}\gamma ,$ and $k_{3}=\cos^{4}\gamma +\sin^{4}\gamma -4\sin^{2}\gamma \cos^{2}\gamma$.

For polycrystals, if two material points $x_{\left(k\right)}$ and $x_{\left(j\right)}$ belong to different grains, the equivalent bond constant $b_{\alpha }$ are calculated by the weighting method.

(21) $$\frac{2}{b_{\alpha }}=\frac{1}{b_{\alpha (k)}}+\frac{1}{b_{\alpha (j)}}(\alpha =1,2)$$

Thus, the PD force density vectors of the interaction between a pair of material points $x_{\left(k\right)}$ and $x_{\left(j\right)}$ in the equation of motion (Equation (1)) can be summed as follows.

(22) $$f=t_{\left(k\right)\left(j\right)}-t_{\left(j\right)\left(k\right)}=\frac{y_{\left(j\right)}-y_{\left(k\right)}}{\left|y_{\left(j\right)}-y_{\left(k\right)}\right|}\left[2ad\frac{\delta }{\left|x_{\left(j\right)}-x_{\left(k\right)}\right|}\Lambda_{\left(k\right)\left(j\right)}\left(\theta_{\left(k\right)}+\theta_{\left(j\right)}\right)+4\delta s_{\left(k\right)\left(j\right)}\left(b_{1}+b_{2}\sin^{2}\varphi \cos^{2}\varphi \right)\right]$$

### 3.3. Surface Correction and Volume Correction 

For uniform discretisation, since the horizon domain is a circle for the two-dimensional plane problem, the material points located at the horizon boundary will have a part of its volume beyond the boundary. The volume correction coefficient $\nu_{\left(j\right)}$ should be introduced to reduce the error [26].

(23) $$\nu_{\left(j\right)}=\left\{\begin{array}{ll} 1 & \left|x_{\left(j\right)}-x_{\left(k\right)}\right|\leq (\delta -\varpi )\\ (\delta +\varpi -\left|x_{\left(j\right)}-x_{\left(k\right)}\right|)/2\varpi & (\delta -\varpi )<\left|x_{\left(j\right)}-x_{\left(k\right)}\right|\leq \delta \\ 0 & otherwise \end{array}\right.$$

where ϖ is half of the discrete spacing of material points, ϖ=∆x/2.

The same issue arises with the material points near the surface/boundary of the computational domain. The integration of the strain energy density in Equation (19) is performed for material points within a complete horizon domain. For a material point $x_{\left(k\right)}$ close to the boundary, it lacks part of its family points because the horizon domain is incomplete, causing the inaccuracy of the simulation results near the boundary. Therefore, the surface correction factors are introduced to deal with this problem when calculating the volume dilatation and the force density vector. The detailed derivation process is shown in Appendix B for the sake of brevity.

The modified volume dilatation and force density vector at point $x_{\left(k\right)}$ are expressed below.

(24) $$\theta_{\left(k\right)}=d\sum_{j=1}^{N}G_{\left(d)(k)(j\right)}\frac{\delta }{\left|x_{\left(j\right)}-x_{\left(k\right)}\right|}\left[\left|y_{\left(j\right)}-y_{\left(k\right)}\right|-\left|x_{\left(j\right)}-x_{\left(k\right)}\right|\right]\Lambda_{\left(k)(j\right)}\nu_{\left(j\right)}V_{\left(j\right)}$$

(25) $$t_{\left(k)(j\right)}=\left[2ad\frac{\delta }{\left|x_{\left(j\right)}-x_{\left(k\right)}\right|}\Lambda_{\left(k)(j\right)}\theta_{\left(k\right)}+2b^{\prime }\delta s_{\left(k)(j\right)}\right]\frac{y_{\left(j\right)}-y_{\left(k\right)}}{\left|y_{\left(j\right)}-y_{\left(k\right)}\right|}\;\mathrm{with}\;b^{\prime }=G_{\left(b)1(k)(j\right)}b_{1}+G_{\left(b)2(k)(j\right)}b_{2}\sin^{2}\varphi \cos^{2}\varphi \frac{y_{\left(j\right)}-y_{\left(k\right)}}{\left|y_{\left(j\right)}-y_{\left(k\right)}\right|}$$

where $G_{\left(d)(k)(j\right)}$ is the surface correction factor for volume dilatation; $G_{\left(b)1(k)(j\right)}$ and $G_{\left(b)2(k)(j\right)}$ are the surface correction factors for the PD material parameters $b_{1}$ and $b_{2}$, respectively.

### 3.4. Damage Model

In the PD damage model for isotropic materials, the damage of the bond between two material points can be determined by either the critical stretch ratio [27] or the critical energy release rate [32]. The critical stretch ratio $s_{0}$ is used in this work, and its expression in the OSB-PD model for two-dimensional problems is:

(26) $$s_{0}=\sqrt{\frac{G_{c}}{\left(bh\delta^{5}+\frac{8}{9}ad^{2}h^{2}\delta^{7}\right)}}$$

where the parameters *a*, *b*, and *d* can be obtained by Equations (18) and (20) to import the influence of orientation angle; $G_{c}$ is the critical energy release rate, which can be estimated by the fracture toughness $K_{Ic}$ and the equivalent elastic modulus $E_{\varphi }$.

(27) $$G_{c}=\left\{\begin{array}{cc} \frac{K_{Ic}^{2}}{E_{\varphi }} & \mathrm{plane}\;\mathrm{stress}\\ \frac{K_{Ic}^{2}}{E_{\varphi }}\left(1-\nu^{2}\right) & \mathrm{plane}\;\mathrm{strain} \end{array}\right.$$

The break of the bond is described by a scalar function $\mu \left(x,x^{\prime },\;t\right)$ correlated with the time *t*. When the stretch ratio between a pair of material points satisfies $s\leq s_{0}$, the bond breaks, that is, $\mu \left(x,x^{\prime },\;t\right)=0$; otherwise, $\mu \left(x,x^{\prime },\;t\right)=1$. In this way, a material point is determined to fail when the bonds between all the family points in its horizon domain and itself are broken. This accumulative damage process can be calculated by the failure function $\psi \left(x,t\right)$ below.

(28) $$\psi \left(x,t\right)=1-\frac{\int_{H_{x}}\mu \left(x,x^{\prime },t\right)dV^{\prime }}{\int_{H_{x}}dV^{\prime }}$$

In addition, the grain boundary strength has an important effect on the fracture behaviours of polycrystalline materials. De Meo et al. [23] defined a grain boundary strength coefficient *β* to study different fracture modes.

(29) $$\beta =\frac{s_{0GB}}{s_{0GI}}$$

where $s_{0GI}$ and $s_{0GB}$ are the critical stretch ratios for the transgranular and intergranular fractures, respectively. When the intergranular fracture is dominated, then β<1, while when the transgranular fracture is dominated, then β>1.

In the following sections, the displacements in static and dynamic problems are solved by the adaptive dynamic relaxation (ADR) and the explicit time integration in Ref. [26], respectively.

## 4. Model Validation and Convergence Analysis 

In this section, the finite element method (FEM) is used to verify the PD model. Through *m*-convergence and *δ*-convergence, the displacements under uniaxial tension and dynamic fracture under biaxial tension are analysed. *δ* is the horizon size, and *m* can present the density of material points in one horizon region with a relationship of *m* = *δ/*∆x (∆x is the spacing of material points). For all the cases in this section, the material is set as *α*-Fe, which is a body-centred cubic lattice, and its elastic stiffness constants are *C*_11_ = 231.4 GPa, *C*_12_ = 134.7 GPa, and *C*_44_ = 116.4 GPa [2]. The fracture toughness is 58.4 $\mathrm{MPa}\cdot \sqrt{m}$ [33]. 

### 4.1. Static Analysis for the Fe Single Crystal with Different Orientations

**Figure 3:**
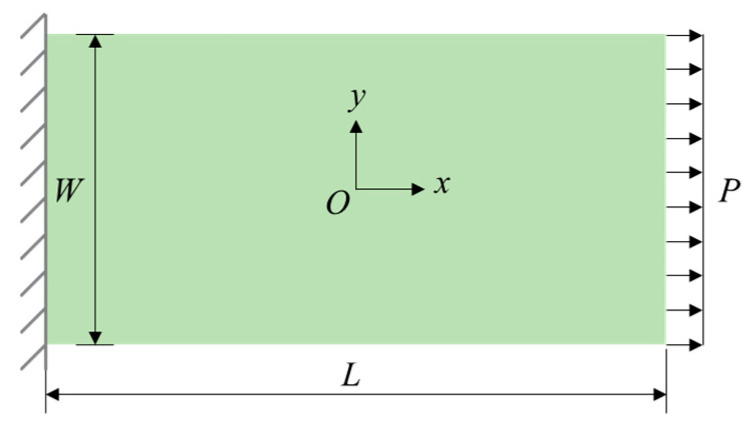
Schematic diagram of a thin rectangular plate under uniaxial tensile loading.

Figure 3 shows a thin rectangular plate under uniaxial tensile loading. It has a length *L* of 20 μm and a width *W* of 10 μm. The thickness *H* is 0.1 μm. On both sides of the plate, three groups of material points are added along the *x*-direction to impose boundary conditions, where the left boundary is completely fixed, and the pressure *P* applied to the right boundary is 150 MPa. 

**Figure 4:**
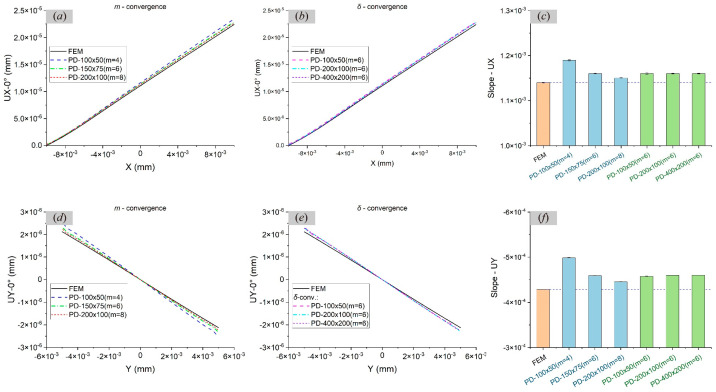
Comparisons of displacement results in single crystals by FEM and PD when *γ* = 0° through (**a**,**d**) *m*-convergence analysis, (**b**,**e**) *δ*-convergence analysis, and (**c**,**f**) the slope of displacement curve.

In order to analyse the effects of the *m* value and *δ* value on the static problem, the displacements UX and UY, respectively, along the centre lines at *y* = 0 and *x* = 0 are studied when the orientation angle *γ* = 0° and compared with FEM results, as shown in Figure 4a,b,d,e. The convergence criterion is $1\times 10^{-8}$. By comparing the slopes of the curves, it can be seen in Figure 4c,f that the increase in the *m* value makes the PD results closer to the FEM results when *δ* is constant. When *m* is constant, decreasing *δ* has little effect on the results. 

**Figure 5:**
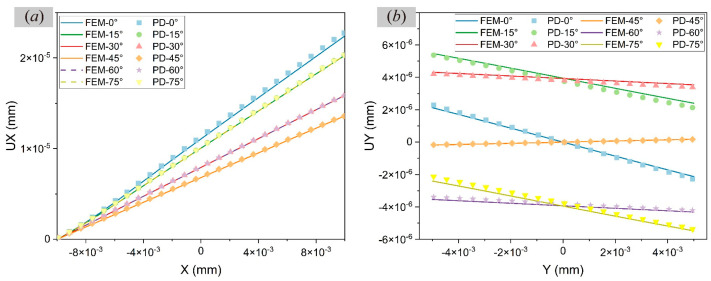
Comparisons of FEM and PD results of displacements (**a**) UX and (**b**) UY at different orientation angles (*γ* = 0°, 15°, 30°, 45°, 60°, 75°).

Comprehensively considering the computational time and accuracy, δ=6Δx and medium mesh size (200 × 100) are taken to compute the uniaxial tensile results at different orientation angles. The displacements at different orientation angles $\gamma \in \left[0,90{}^{\circ}\right]$ are simulated at an interval of 15°. As shown in Figure 5, the PD results are consistent with the corresponding FEM results at each orientation angle, indicating the effectiveness of the proposed PD model in simulating the static problem of a single cubic crystal with a given orientation angle. 

### 4.2. Static Analysis for Polycrystalline Materials 

**Figure 6:**
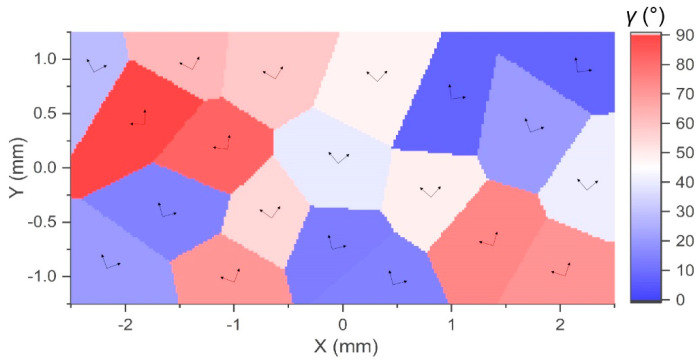
Voronoi diagram of a polycrystalline rectangular plate containing 20 grains and the corresponding grain orientations.

**Figure 7:**
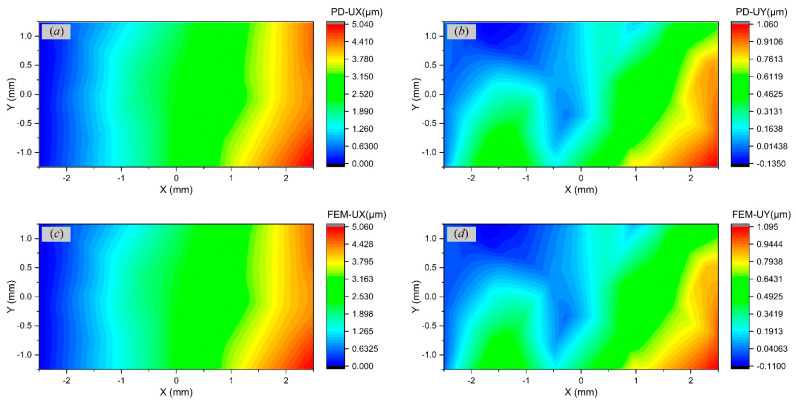
Comparisons of displacement contours in a polycrystalline rectangular plate by (**a**,**b**) PD and (**c**,**d**) FEM.

The polycrystalline microstructure is generated by the Voronoi tessellation method. Voronoi seeds are calculated by the pseudo-random Sobel sequence to obtain the microstructure with relatively uniform grain size and distribution. Figure 6 shows a polycrystalline rectangular thin plate with 20 randomly oriented grains. The range of the orientation angles γ is $\left[0,90{}^{\circ}\right]$. The computational domain has a length *L* of 5 mm and a width *W* of 2.5 mm. The plate thickness *H* is 0.01 mm. The numbers of material points in the *x*- and *y*-directions are 240 and 120, respectively. The horizon size δ is 6Δx. The loading condition is the same as the one for single crystals in Section 4.1. Figure 7 illustrates the displacement contours in the *x*- and *y*-directions from PD and FEM simulations, respectively. The convergence criterion is $1\times 10^{-8}$. The results of the two methods are almost consistent, which validates the effectiveness of the proposed PD method in calculating the static behaviours of polycrystalline materials. 

### 4.3. Convergence Analysis of Dynamic Fracture of Polycrystalline Materials 

Many studies have shown that mesh size and horizon size can significantly influence the crack propagation path in polycrystalline materials [23,27], but most size-independent convergence analyses are focused on static problems. It is important to obtain the convergence results of a crack propagation path for predicting and controlling the dynamic fracture of polycrystalline materials. Like static analysis, *m*-convergence and *δ*-convergence are also good methods to study the influence of meshing and horizon size on the crack propagation path. Three GBC values of 0.5, 2, and 1 are selected to represent intergranular, transgranular, and mixed fracture modes.

**Figure 8:**
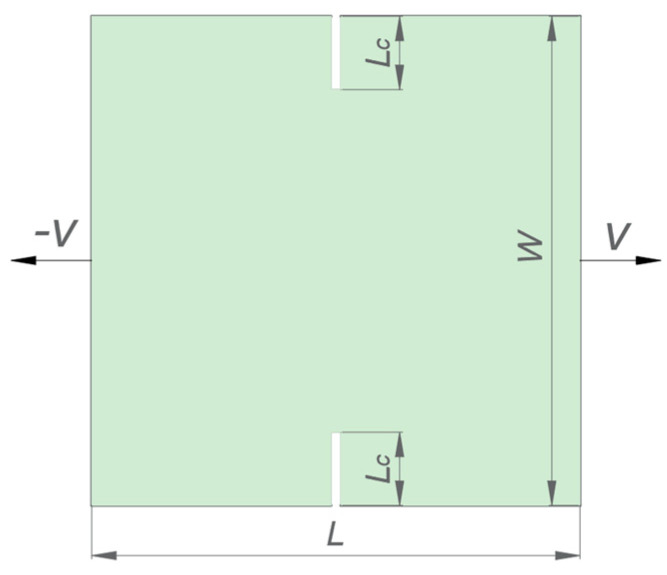
Schematic diagram of the polycrystalline rectangular plate with two preset cracks under biaxial tensile loading.

Figure 8 shows a 5 mm × 5 mm rectangular plate with preset cracks at the top and the bottom. It contains 100 grains with random orientation angles within [−45°, 45°]. The length of each crack *Lc* is 0.4 mm. The left and right sides of the plate are subjected to a rapid tensile velocity *V* of 15 m/s in opposite directions. 

**Figure 9:**
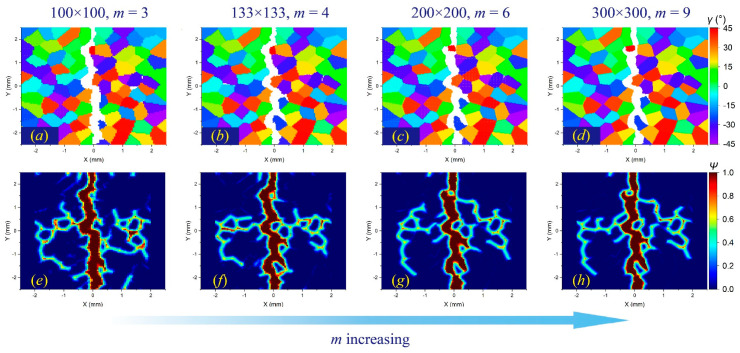
The *m*-convergence analysis of crack propagation paths in polycrystalline materials containing 100 grains at time step 3000 when GBC = 0.5. From left to right, when *δ* remains constant and *m* increases, (**a**–**d**) the distributions of failed material points in the polycrystalline structure and (**e**–**h**) the corresponding damage coefficient contours are shown, respectively.

**Figure 10:**
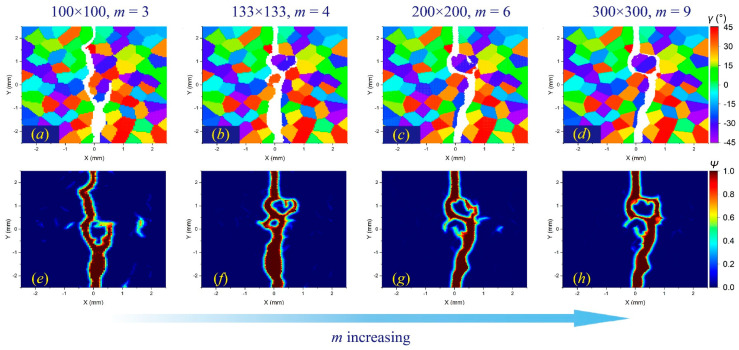
The *m*-convergence analysis of crack propagation paths in polycrystalline materials containing 100 grains at time step 3000 when GBC = 1. From left to right, when *δ* remains constant and *m* increases, (**a**–**d**) the distributions of failed material points in the polycrystalline structure and (**e**–**h**) the corresponding damage coefficient contours are shown, respectively.

**Figure 11:**
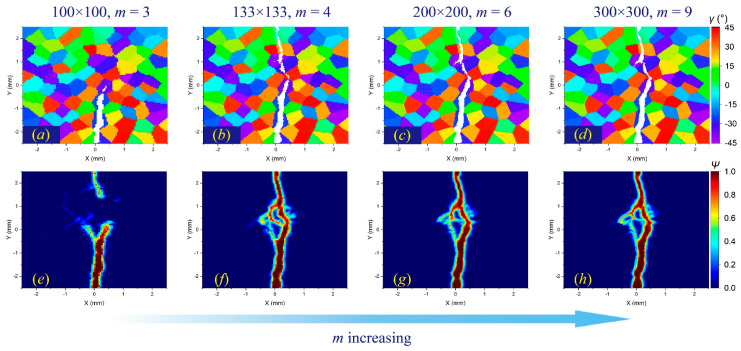
The *m*-convergence analysis of crack propagation paths in polycrystalline materials containing 100 grains at time step 3000 when GBC = 2. From left to right, when *δ* remains constant and *m* increases, (**a–d**) the distributions of failed material points in the polycrystalline structure and (**e–h**) the corresponding damage coefficient contours are shown, respectively.

Figure 9, Figure 10 and Figure 11 illustrate the *m*-convergence results of different GBC values (fracture modes) at the time steps of 3000 (3 μs). It can be seen that when *m* is higher than 6, the changes in the crack propagation path in the three fracture modes decrease with the increase in the *m* value and tend to be converged. 

**Figure 12:**
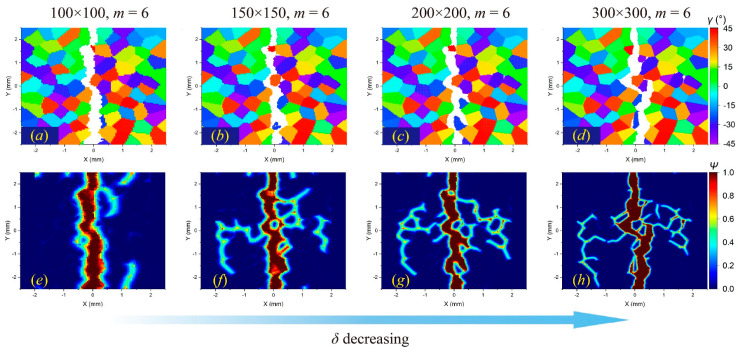
The *δ*-convergence analysis of crack propagation paths in polycrystalline materials containing 100 grains at time step 3000 when GBC = 0.5. From left to right, when *m* remains constant and *δ* decreases, (**a**–**d**) the distributions of failed material points in the polycrystalline structure and (**e**–**h**) the corresponding damage coefficient contours are shown, respectively.

**Figure 13:**
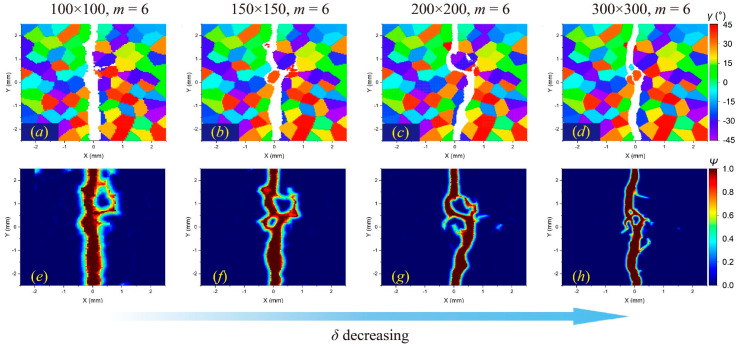
The *δ*-convergence analysis of crack propagation paths in polycrystalline materials containing 100 grains at time step 3000 when GBC = 1. From left to right, when *m* remains constant and *δ* decreases, (**a**–**d**) the distributions of failed material points in the polycrystalline structure and (**e**–**h**) the corresponding damage coefficient contours are shown, respectively.

In comparison, the *δ*-convergence results show more uncertainties. The change in the *δ* value affects the crack propagation path in the intergranular fracture more than in the transgranular fracture, as shown in Figure 12 and Figure 13. The decrease in *δ* indicates a reduction in the integral area when calculating the stretch ratio, resulting in the difference in damage determination. This difference will have a more prominent influence on the boundary of adjacent grains with large differences in the orientation angle, which may lead to different crack propagation paths.

**Figure 14:**
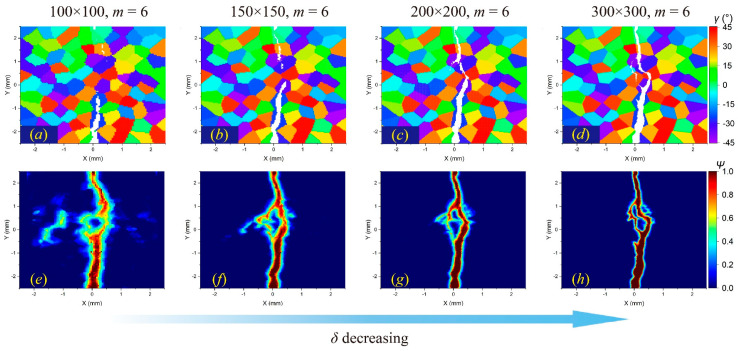
The *δ*-convergence analysis of crack propagation paths in polycrystalline materials containing 100 grains at time step 3000 when GBC = 2. From left to right, when *m* remains constant and *δ* decreases, (**a**–**d**) the distributions of failed material points in the polycrystalline structure and (**e**–**h**) the corresponding damage coefficient contours are shown, respectively.

In addition, when the horizon size is higher than the size of a single grain, the results will also be different. For a meshing size of 100 × 100, when *m* = 6, there are 112 material points in a complete horizon domain, while the average number of material points in one grain is about 100. For an intergranular fracture, the number of local microcracks will be significantly reduced (Figure 12e), while for a transgranular fracture, local damage may increase (Figure 14e). 

Overall, the horizon size (*δ*) has a greater impact on crack propagation paths than the density of material points in one horizon region (*m*). When conducting dynamic fracture analysis of polycrystalline materials by the PD method, it is suggested to determine a proper *δ* value first and then increase the *m* value to achieve a faster convergence result of the crack propagation path. It is also necessary to ensure that the number of material points in a single grain is larger than that in a horizon region.

## 5. Applications on Dynamic Fractures 

This section mainly studies and discusses the applications of the proposed model in two aspects. One is the effect of crystal orientations on the crack initiation and propagation of single-crystal materials with different notch shapes and sizes. The other is the effect of the degree of anisotropy in polycrystalline materials on propagation growth paths under different fracture modes.

### 5.1. Single-Crystal Materials with Different Notch Shapes and Sizes

**Figure 15:**
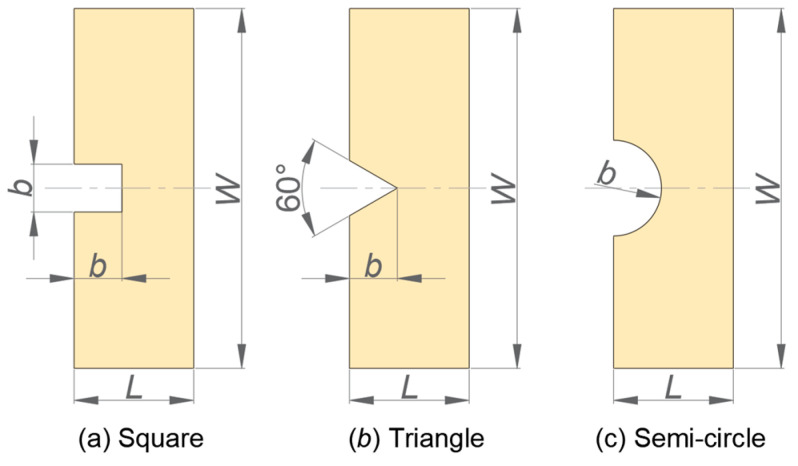
Schematic diagram of a single-crystal rectangular plate with different notch shapes including (**a**) square, (**b**) equilateral triangle, and (**c**) semi-circle.

Notches of various kinds exist in part/component design, such as chamfer, fillet, and thread. However, several studies have indicated that the stress concentration around the notch tip would significantly reduce the load capacity of parts/components [34]. For a single crystal, it has been found that the orientation difference between the notch front and crystal also makes the crack propagation direction change [3]. In order to study the effects of notch shape and size on the crack propagation path, three notch shapes including square, equilateral triangle, and semi-circle are designed on a rectangular plate, as shown in Figure 15. Dimension *b* is the side length, height, and radius of the square, triangle, and semi-circle, respectively. 

The plate’s length, width, and thickness are 5 mm, 15 mm, and 0.1 mm, respectively. The single cubic crystal is silicon (C_11_ = 165.6 GPa, C_12_ = 63.9 GPa, C_44_ = 79.5 GPa). The fracture toughness is 2 $\mathrm{MPa}\cdot \sqrt{m}$ for all orientation angles because it has little dependence on the tensile direction in the same crystal plane [3]. The velocity imposed on the top and the bottom side of the plate is ±0.5 m/s. The orientation angles are 0°, 30°, 45°, and 60°. The value of dimension *b* is 0.5, 1, and 1.5 mm. 

**Figure 16:**
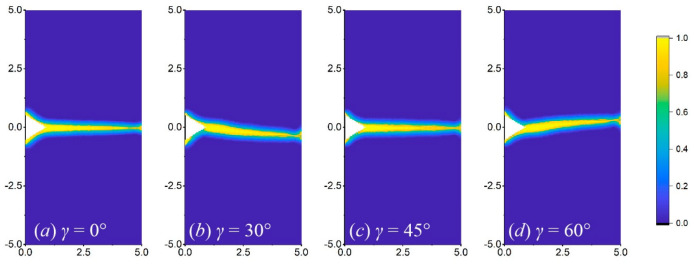
Crack propagation paths of the plate with a triangular notch at different orientation angles of (**a**) 0°, (**b**) 30°, (**c**) 45°, and (**d**) 60°, when *b* = 1 mm.

**Figure 17:**
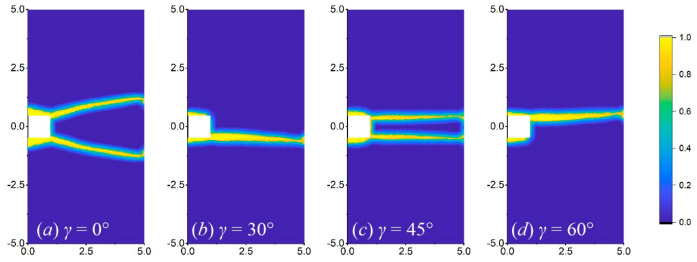
Crack propagation paths of the plate with a square notch at different orientation angles of (**a**) 0°, (**b**) 30°, (**c**) 45°, and (**d**) 60°, when *b* = 1 mm.

**Figure 18:**
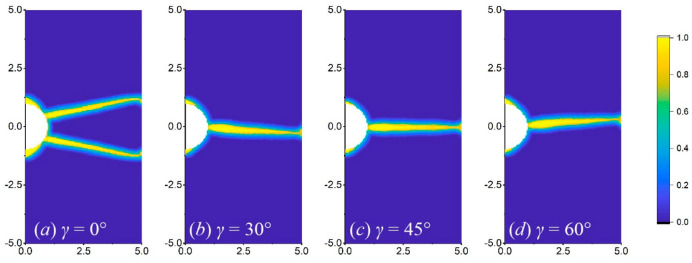
Crack propagation paths of the plate with a semi-circular notch at different orientation angles of (**a**) 0°, (**b**) 30°, (**c**) 45°, and (**d**) 60°, when *b* = 1 mm.

Figure 16, Figure 17 and Figure 18 show the crack propagation paths of equilateral triangular, square, and semi-circular notches at different orientation angles when *b* is 1 mm. Under the same working conditions, the equilateral triangular notch is less likely to bifurcate than the others. At the orientation angles of 30° and 60°, the crack propagation paths of the three notch shapes are obviously shifted relative to the horizontal line and are basically symmetrical, which reflects the force shift caused by the orientation angle. For the square notch, when the orientation angles are 0° and 45°, the two right angles induce two crack routes (Figure 17a,c), while at the crystal orientation of 30° and 60°, the crack only occurs from one right angle (Figure 17b,d). As for the semi-circular notch, bifurcation is more likely to occur at a crystal orientation of 0° than at other angles, as shown in Figure 18.

**Figure 19:**
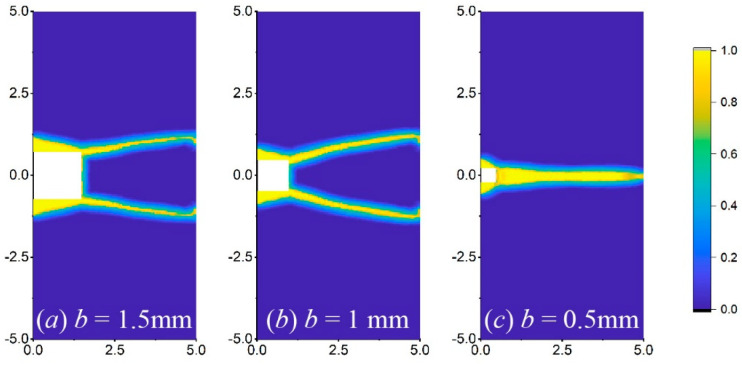
Crack propagation paths of the plate with a square notch at different dimensions of (**a**) 1.5 mm, (**b**) 1 mm, and (**c**) 0.5 mm when *γ* = 0°.

Additionally, the notch dimension has a greater effect on the crack propagation path of the square notch, as shown in Figure 19. At the crystal orientation of 0°, when the notch dimension gradually decreases, the number of crack routes reduces from two to one. However, it has smaller changes in the pattern and number of crack routes in the triangular and semi-circular cases, so it is not shown for simplicity.

**Figure 20:**
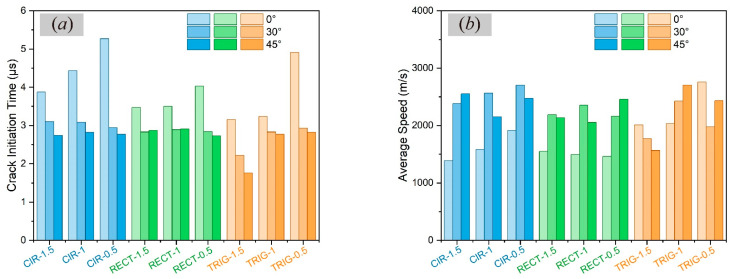
Results of (**a**) crack initiation time and (**b**) average propagation speed in single-crystal materials with three notch shapes and sizes under different orientation angles (CIR—semi-circle, RECT—square, TRIG—triangle. The numbers 1.5, 1, and 0.5 are the values of **b**).

The crack initiation time and the average propagation speed are also studied. As shown in Figure 20a, when the crystal orientation is 0°, that is, the loading direction is parallel or vertical to the crystal orientation, the crack initiation is the slowest compared with the other angles for all notch shapes. As the notch size increases, crack initiation occurs faster. Among the three shapes, the semi-circular notch takes the longest time to initiate cracks.

However, the later occurrence of the crack does not mean that the crack propagation speed will be slow, as shown in Figure 20b. When *γ* = 0°, the propagation speeds of the semi-circular and square notches are the slowest, while the speed of the triangular notch is the fastest at the size of 1.5 mm and 0.5 mm. For different notch shapes, the semi-circular notch with a larger size has the lowest speed at *γ* = 0°.

In short, the numerical results showed that the crack propagation path of single crystals is sensitive to crystal orientation, notch shape, and size. To be more specific, semi-circular notches are the most resistant to fracture in most cases, which is why rounded corners are often used in structural design to reduce the stress concentration at edges and corners. Rectangular and triangular notches are more likely to crack because of stress concentration caused by sharp corners, which can be improved by modifying the tip to a rounded corner [35]. 

In addition, the change in crystal orientation results in a change in the crack bifurcation, the number of crack propagation paths, and the direction deviation. It needs to be analysed in combination with the material property and structure. For single-crystal silicon, experimental results showed that cracks occur along several specific cleavage planes. For example, the fracture plane of Si(100) sheets after biaxial tensile loading is mainly along the plane {110} [3]. The results in Figure 18a,c may be closer to this experimental trend. However, further improvements in the theoretical part are required for the fracture with specific cleavage planes.

### 5.2. Effects of Anisotropy Degree in Polycrystalline Materials

**Figure 21:**
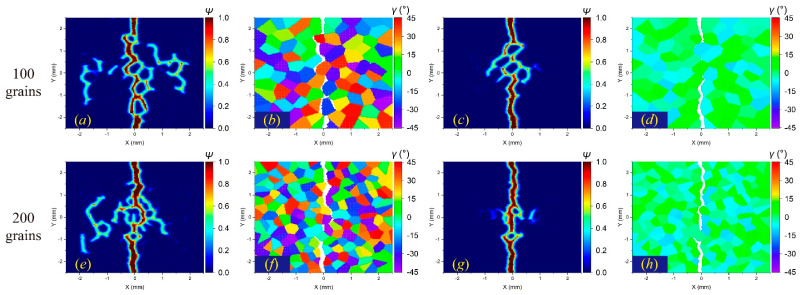
Crack distributions in the polycrystalline plate at time step 2000 when GBC = 0.5: (**a**,**b**) 100 grains with high anisotropy degree, (**c**,**d**) 100 grains with low anisotropy degree, (**e**,**f**) 200 grains with high anisotropy degree, and (**g**,**h**) 200 grains with low anisotropy degree.

**Figure 22:**
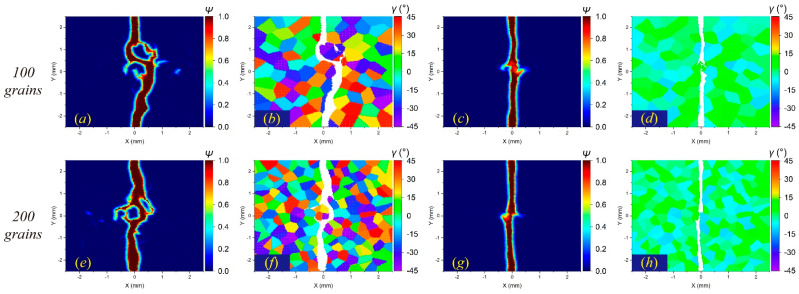
Crack distributions in the polycrystalline plate at time step 3000 when GBC = 1: (**a**,**b**) 100 grains with high anisotropy degree, (**c**,**d**) 100 grains with low anisotropy degree, (**e**,**f**) 200 grains with high anisotropy degree, and (**g**,**h**) 200 grains with low anisotropy degree.

**Figure 23:**
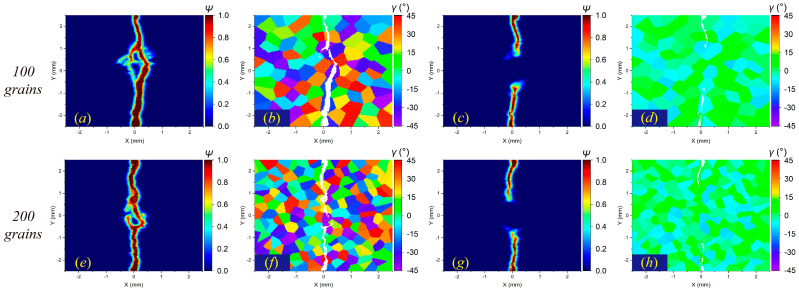
Crack distributions in the polycrystalline plate at time step 3000 when GBC = 2: (**a**,**b**) 100 grains with high anisotropy degree, (**c**,**d**) 100 grains with low anisotropy degree, (**e**,**f**) 200 grains with high anisotropy degree, and (**g**,**h**) 200 grains with low anisotropy degree.

The anisotropy degree caused by grain orientations has a significant influence on the fracture properties of polycrystalline materials. Based on the results in Section 4.3, the plate with a crystal orientation range of [−10°,10°] and a grain number of 200 is further simulated for comparison. The crack distribution results under different fracture modes are shown in Figure 21, Figure 22 and Figure 23.

Figure 21 shows that when intergranular fracture is dominant, the reduction in the anisotropy degree will make the microcrack around the main propagation routes disappear to a large extent. When the two fracture modes are not distinguished, the decrease in the anisotropy degree reduces the bifurcation in the crack propagation path, even approaching a straight line, as shown in Figure 22. When transgranular fracture dominates, the crack propagation with a low degree of anisotropy is significantly slower than that with a high degree of anisotropy (see Figure 23). These simulation results indicate that cracks in polycrystalline materials tend to occur at locations with larger differences in orientation angles, which has also been found in experimental studies [8].

Practically, the grain orientation and size of a polycrystalline material may be random or regular, depending on the manufacturing method. In the application of the model, its fracture toughness and crack propagation path can be calibrated through small amounts of experiments. Then, a more fracture-resistant grain structure and orientation can be obtained through optimisation design in simulations to guide the structural design and manufacturing of polycrystalline materials. 

## 6. Conclusions

In view of the shortcomings of existing PD models in simulating crystal orientation, this work developed an improved OSB-PD model to simulate the granular fractures of cubic crystals involving arbitrary crystal orientations and delivered a complete derivation process and expressions of correction factors. Two numerical experiments are used to study and discuss the effect of crystal orientation on crack propagation. The main findings and innovations of this work are summarised as follows.

(1) The periodic characteristics of the equivalent elastic modulus of cubic crystals are introduced into the PD parameters to achieve the simulation of arbitrary crystal orientations.
(2) Convergence analysis is carried out in static and dynamic problems to obtain a proper density of material points in one horizon region (*m* value) and horizon size (*δ* value) to ensure computational effectiveness and accuracy. For a static problem, the *m* value has a greater effect on convergence than the *δ* value, while for a dynamic fracture problem, the *δ* value influences the crack propagation path more than the *m* value, especially in the intergranular fracture mode.
(3) For convergence analysis on dynamic problems, a regulating strategy to obtain the converged and accurate results of crack propagation paths is proposed as follows: select an appropriate horizon size first and then increase the *m* value until the accuracy is satisfied.
(4) In the numerical examples, the influence of crystal orientation on single-crystal materials with different notch shapes and sizes is mainly reflected in bifurcations, numbers, and propagation path directions of cracks. Under biaxial tensile loading, the single crystal with a semi-circular notch is more resistant to fracture than the crystal with square or triangular notches in most cases.
(5) For polycrystalline materials, the decrease in the degree of grain anisotropy reduces microcracks in intergranular fracture and the crack propagation rate in transgranular fracture.

Therefore, the OSB-PD model proposed in this work can simulate the granular fracture behaviours of cubic crystals with arbitrary orientation angles and evaluate the fracture properties of single crystals and polycrystalline materials. The size parameters obtained by convergence analysis can ensure the accuracy of the crack propagation path and provide a reference for the microstructure design and manufacturing of polycrystalline materials. However, improvements are still needed for materials with specific fracture cleavage planes and three-dimensional problems.

## Data Availability

The original contributions presented in the study are included in the article, further inquiries can be directed to the corresponding author.

## Appendix A. Derivation of the PD Material Parameters for Cubic Crystals

The PD material parameters for cubic crystals are derived in the same way as those for isotropic materials and composite laminates by comparing the volume dilatation and strain energy density in CCM theory under simple loading conditions. Here, uniaxial tension along the crystal orientation, simple shear, and biaxial tension are considered on a cubic crystal rectangular plate.

- **First Loading**—Uniaxial tensile in the direction of crystal orientation: $\varepsilon_{11}=\zeta ,\varepsilon_{22}=0$.

The magnitude of the relative position vector in the deformed configuration is:

(A1) $$\left|y_{\left(j\right)}-y_{\left(k\right)}\right|=\left[1+\left(\cos^{2}\phi \right)\zeta \right]\left|x_{\left(j\right)}-x_{\left(k\right)}\right|.$$

(1) Volume dilatation.

(A2) $$\mathrm{CCM}\;\mathrm{theory}:\;\theta_{\left(k\right)}^{CCM}=\zeta$$

(A3) $$\mathrm{PD}\;\mathrm{theory}:\;\theta_{\left(k\right)}^{PD}=d\int_{H}\frac{\delta }{\xi }(\left[1+(\cos^{2}\phi )\zeta \right]\xi -\xi )dH=\frac{\pi dh\delta^{3}\zeta }{2}$$

Let $\theta_{\left(k\right)}^{PD}=\theta_{\left(k\right)}^{CCM}$, so that

(A4) $$d=\frac{2}{\pi h\delta^{3}}.$$

(2) Strain energy density.

(A5) $$\mathrm{CCM}\;\mathrm{theory}:\;W_{\left(k\right)}^{CCM}=\frac{1}{2}R_{11}\zeta^{2}$$

(A6)
$$\begin{array}{ll} \mathrm{PD}\;\mathrm{theory}: & \\ \;\;\;W_{\left(k\right)}^{PD}=a\zeta^{2}+b_{1} & \int_{H}\frac{\delta }{\xi }(\left[1+\left(\cos^{2}\phi \right)\zeta \right]\xi -\xi {)}^{2}dH+b_{2}\int_{H}\frac{\delta }{\xi }\left(\sin^{2}\varphi \cos^{2}\varphi \right)(\left[1+\left(\cos\phi \sin\phi \right)\zeta \right]\xi -\xi {)}^{2}dH\\ & =a\zeta^{2}+b_{1}h\int_{0}^{\delta }\int_{0}^{2\pi }\frac{\delta }{\xi }(\left[1+\left(\cos^{2}\phi \right)\zeta \right]\xi -\xi {)}^{2}\xi d\phi d\xi \\ & +b_{2}h\int_{0}^{\delta }\int_{0}^{2\pi }\frac{\delta }{\xi }\sin^{2}\left(\phi -\gamma \right)\cos^{2}\left(\phi -\gamma \right)(\left[1+(\cos^{2}\phi )\zeta \right]\xi -\xi {)}^{2}\xi d\phi d\xi \\ & =a\zeta^{2}+\left(\frac{\pi h\delta^{4}\zeta^{2}}{4}\right)b_{1}+\left(\frac{\left(38\pi k_{1}+5\pi k_{3}\right)h\delta^{4}\zeta^{2}}{192}\right)b_{2} \end{array}$$

- **Second Loading**—Simple shear: $\gamma_{12}=\zeta$.

The magnitude of the relative position vector in the deformed configuration is:

(A7) $$\left|y_{\left(j\right)}-y_{\left(k\right)}\right|=\left[1+\left(\sin\phi \cos\phi \right)\zeta \right]\left|x_{\left(j\right)}-x_{\left(k\right)}\right|.$$

(1) Volume dilatation.

(A8) $$\theta_{\left(k\right)}^{CCM}=0$$

(2) Strain energy density.

(A9) $$\mathrm{CCM}\;\mathrm{theory}:\;W_{\left(k\right)}^{CCM}=\frac{1}{2}R_{44}\zeta^{2}$$

(A10)
$$\begin{array}{ll} \mathrm{PD}\;\mathrm{theory}: & \\ \;W_{\left(k\right)}^{PD}=a\left(0\right) & +b_{1}\int_{H}\frac{\delta }{\xi }(\left[1+(\cos\phi \sin\phi )\zeta \right]\xi -\xi {)}^{2}dH+b_{2}\int_{H}\frac{\delta }{\xi }\left(\sin^{2}\varphi \cos^{2}\varphi \right)(\left[1+(\cos\phi \sin\phi )\zeta \right]\xi -\xi {)}^{2}dH\\ & =a\left(0\right)+b_{1}h\int_{0}^{\delta }\int_{0}^{2\pi }\frac{\delta }{\xi }(\left[1+(\cos\phi \sin\phi )\zeta \right]\xi -\xi {)}^{2}\xi d\phi d\xi \\ & +b_{2}h\int_{0}^{\delta }\int_{0}^{2\pi }\frac{\delta }{\xi }\sin^{2}\left(\phi -\gamma \right)\cos^{2}\left(\phi -\gamma \right)(\left[1+(\cos\phi \sin\phi )\zeta \right]\xi -\xi {)}^{2}\xi d\phi d\xi \\ & =\left(\frac{\pi h\delta^{4}\zeta^{2}}{12}\right)b_{1}+\left(\frac{\left(10\pi k_{1}+3\pi k_{3}\right)h\delta^{4}\zeta^{2}}{192}\right)b_{2} \end{array}$$

- **Third loading**—biaxial tensile: $\varepsilon_{11}=\zeta ,\varepsilon_{22}=\zeta$.

The magnitude of the relative position vector in the deformed configuration is:

(A11) $$\left|y_{\left(j\right)}-y_{\left(k\right)}\right|=\left[1+\zeta \right]\left|x_{\left(j\right)}-x_{\left(k\right)}\right|.$$

(1) Volume dilatation.

(A12) $$\theta_{\left(k\right)}^{CCM}=2\zeta$$

(2) Strain energy density.

(A13) $$\mathrm{CCM}\;\mathrm{theory}:\;W_{\left(k\right)}^{CCM}=\left(R_{11}+R_{12}\right)\zeta^{2}$$

(A14)
$$\begin{array}{ll} \mathrm{PD}\;\mathrm{theory}: & \\ & W_{\left(k\right)}^{PD}=4a\zeta^{2}+b_{1}\int_{H}\frac{\delta }{\xi }(\left[1+\zeta \right]\xi -\xi {)}^{2}dH+b_{2}\int_{H}\frac{\delta }{\xi }\left(\sin^{2}\varphi \cos^{2}\varphi \right)(\left[1+\zeta \right]\xi -\xi {)}^{2}dH\\ & =4a\zeta^{2}+b_{1}h\int_{0}^{\delta }\int_{0}^{2\pi }\frac{\delta }{\xi }(\left[1+\zeta \right]\xi -\xi {)}^{2}\xi d\phi d\xi \\ & +b_{2}h\int_{0}^{\delta }\int_{0}^{2\pi }\frac{\delta }{\xi }\sin^{2}\left(\phi -\gamma \right)\cos^{2}\left(\phi -\gamma \right)(\left[1+\zeta \right]\xi -\xi {)}^{2}\xi d\phi d\xi \\ & =4a\zeta^{2}+\left(\frac{2\pi h\delta^{4}\zeta^{2}}{3}\right)b_{1}+\left(\frac{\left(6\pi k_{1}+\pi k_{3}\right)h\delta^{4}\zeta^{2}}{12}\right)b_{2} \end{array}$$

Let $W_{\left(k\right)}^{PD}=W_{\left(k\right)}^{CCM}$ in the three loading conditions, then the equation sets can be obtained as follows.

(A15) $$\left\{\begin{array}{l} \left(\frac{\pi h\delta^{4}}{12}\right)b_{1}+\frac{\left(10\pi k_{1}+3\pi k_{3}\right)h\delta^{4}}{192}b_{2}=\frac{1}{2}R_{44}\\ a+\left(\frac{\pi h\delta^{4}}{4}\right)b_{1}+\frac{\left(38\pi k_{1}+5\pi k_{3}\right)h\delta^{4}}{192}b_{2}=\frac{1}{2}R_{11}\\ 4a+\left(\frac{2\pi h\delta^{4}}{3}\right)b_{1}+\frac{\left(6\pi k_{1}+\pi k_{3}\right)h\delta^{4}}{12}b_{2}=(R_{11}+R_{12}) \end{array}\right.$$

The other three PD material parameters are solved:

(A16) $$\begin{array}{l} a=\frac{1}{2}\left(R_{12}-R_{44}\right),\\ b_{1}=\frac{3}{2\pi h\delta^{4}k}\left[\left(10\pi k_{1}+3\pi k_{3}\right)(R_{11}-R_{12})-2\left(14\pi k_{1}+\pi k_{3}\right)R_{44}\right]\;\mathrm{and}\\ b_{2}=\frac{24\left[2R_{44}-(R_{11}-R_{12})\right]}{h\delta^{4}k}. \end{array}$$

where $k=\pi k_{3}-2\pi k_{1}$, $k_{1}=\sin^{2}\gamma \cos^{2}\gamma ,$ and $k_{3}=\cos^{4}\gamma +\sin^{4}\gamma -4\sin^{2}\gamma \cos^{2}\gamma$.

## Appendix B. Surface Correction Factors

By imposing a constant displacement gradient, the uniaxial tensile loadings in *x*- and *y*-directions are conducted on a rectangular plate: $\partial u_{x}^{*}/\partial x_{\alpha }=\zeta (\alpha =1,2)$.

The volume dilatations in PD and CCM theory are

(A17) $$\theta_{\left(k\right)}^{PD}=d\sum_{j=1}^{N}\frac{\delta }{\left|x_{\left(j\right)}-x_{\left(k\right)}\right|}(\left|y_{\left(j\right)}-y_{\left(k\right)}\right|-\left|x_{\left(j\right)}-x_{\left(k\right)}\right|)\Lambda_{\left(k)(j\right)}V_{\left(j\right)}$$

(A18) $$\theta_{\left(k\right)}^{CCM}=\zeta$$

The surface correction factor for the volume dilatation is

(A19) $$D_{\alpha (i)}=\frac{\zeta }{d\delta \sum_{j=1}^{N}s_{\left(k)(j\right)}\Lambda_{\left(k)(j\right)}V_{\left(j\right)}}$$

The strain energy density in PD theory is

(A20) $$W_{\alpha }^{PD}[x_{\left(k\right)}]=W_{\alpha \theta }^{PD}[x_{\left(k\right)}]+W_{\alpha 1}^{PD}[x_{\left(k\right)}]+W_{\alpha 2}^{PD}[x_{\left(k\right)}]$$

where $W_{\alpha \theta }^{PD}[x_{\left(k\right)}]$, $W_{\alpha 1}^{PD}\left[x_{\left(k\right)}\right],$ and $W_{\alpha 2}^{PD}[x_{\left(k\right)}]$ are the contributions from volume strain ($\theta_{\left(k\right)}^{PD}$), the isotropic base ($b_{1}$), and the anisotropic part (${b_{2}\sin}^{2}\varphi \cos^{2}\varphi$).

In Appendix A, the strain energy density under the uniaxial tensile loading is

(A21) $$\begin{array}{ll} W_{\alpha }^{PD}[x_{\left(i\right)}]= & a{\left[\theta_{\left(k\right)}^{PD}\right]}^{2}+b_{1}\sum_{j=1}^{M}\frac{\delta }{\left|x_{\left(j\right)}-x_{\left(k\right)}\right|}(\left|y_{\left(j\right)}-y_{\left(k\right)}\right|-\left|x_{\left(j\right)}-x_{\left(k\right)}\right|{)}^{2}V_{\left(j\right)}\\ & +b_{2}\sum_{j=1}^{J}\sin^{2}\varphi \cos^{2}\varphi \frac{\delta }{\left|x_{\left(j\right)}-x_{\left(k\right)}\right|}(\left|y_{\left(j\right)}-y_{\left(k\right)}\right|-\left|x_{\left(j\right)}-x_{\left(k\right)}\right|{)}^{2}V_{\left(j\right)} \end{array}$$

Thus, the three components can be separated as

(A22) $$\begin{array}{l} W_{\alpha \theta }^{PD}[x_{\left(k\right)}]=a{\left[\theta_{\left(k\right)}^{PD}\right]}^{2}\\ W_{\alpha 1}^{PD}\left[x_{\left(k\right)}\right]=b_{1}\delta \sum_{j=1}^{M}\frac{(\left|y_{\left(j\right)}-y_{\left(k\right)}\right|-\left|x_{\left(j\right)}-x_{\left(k\right)}\right|{)}^{2}}{\left|x_{\left(j\right)}-x_{\left(k\right)}\right|}V_{\left(j\right)}\\ W_{\alpha 2}^{PD}[x_{\left(k\right)}]=b_{2}\delta \sum_{j=1}^{J}\sin^{2}\varphi \cos^{2}\varphi \frac{(\left|y_{\left(j\right)}-y_{\left(k\right)}\right|-\left|x_{\left(j\right)}-x_{\left(k\right)}\right|{)}^{2}}{\left|x_{\left(j\right)}-x_{\left(k\right)}\right|}V_{\left(j\right)} \end{array}$$

At the same time, the strain energy density in CCM theory can also be divided into three parts.

(A23) $$W_{\alpha }^{CCM}[x_{\left(k\right)}]=\frac{1}{2}Q_{\alpha \alpha }\zeta^{2}=W_{\alpha \theta }^{CCM}[x_{\left(k\right)}]+W_{\alpha 1}^{CCM}[x_{\left(k\right)}]+W_{\alpha 2}^{CCM}[x_{\left(k\right)}]$$

By substituting the PD material parameters into it, the components can be expressed as follows.

(A24) $$\begin{array}{l} W_{\alpha \theta }^{CCM}[x_{\left(k\right)}]=a\zeta^{2}\\ W_{\alpha 1}^{CCM}[x_{\left(k\right)}]=\frac{\pi h\delta^{4}\zeta^{2}}{4}b_{1}\\ W_{\alpha 2}^{CCM}[x_{\left(k\right)}]=\frac{\left(38\pi k_{1}+5\pi k_{3}\right)h\delta^{4}\zeta^{2}}{192}b_{2} \end{array}$$

The surface correction factors for the isotropic base ($b_{1}$) and anisotropic part ($b_{2}\sin^{2}\varphi \cos^{2}\varphi$) are calculated by

(A25) $$S_{1(k)}=\frac{W_{\alpha 1}^{CCM}[x_{\left(k\right)}]}{W_{\alpha 1}^{PD}[x_{\left(k\right)}]}=\frac{\frac{\pi h\delta^{4}}{4}b_{1}}{b_{1}\sum_{j=1}^{M}\frac{\delta }{\left|x_{\left(j\right)}-x_{\left(k\right)}\right|}(\left|y_{\left(j\right)}-y_{\left(k\right)}\right|-\left|x_{\left(j\right)}-x_{\left(k\right)}\right|{)}^{2}V_{\left(j\right)}}\times \zeta^{2}$$

(A26) $$S_{2(k)}=\frac{W_{\alpha 2}^{CCM}[x_{\left(k\right)}]}{W_{\alpha 2}^{PD}[x_{\left(k\right)}]}=\frac{\frac{\left(38\pi k_{1}+5\pi k_{3}\right)h\delta^{4}}{192}b_{2}}{b_{2}\sum_{j=1}^{J}\sin^{2}\varphi \cos^{2}\varphi \frac{\delta }{\left|x_{\left(j\right)}-x_{\left(k\right)}\right|}(\left|y_{\left(j\right)}-y_{\left(k\right)}\right|-\left|x_{\left(j\right)}-x_{\left(k\right)}\right|{)}^{2}V_{\left(j\right)}}$$

According to these factors, the vectors of the surface correction factor for the volume dilatation and the integration of the strain energy density at the material point $x_{\left(k\right)}$ are

(A27) $$g_{\left(d)(k\right)}[x_{\left(k\right)}]={\left\{g_{1(d)}[x_{\left(k\right)}],g_{2(d)}[x_{\left(k\right)}]\right\}}^{T}={\left\{D_{1(k)},D_{2(k)}\right\}}^{T}$$

(A28) $$g_{\left(b)l(k\right)}[x_{\left(k\right)}]={\left\{g_{1(b)l}[x_{\left(k\right)}],g_{2(b)l}[x_{\left(k\right)}]\right\}}^{T}={\left\{S_{1l(k)},S_{2l(k)}\right\}}^{T}$$

where l=1 and 2 for $b_{1}$ and $b_{2}$, respectively.

Typically, the surface correction factors at $x_{\left(k\right)}$ and $x_{\left(j\right)}$ are different. Thus, the average value of them is used.

(A29) $${\bar{g}}_{\left(d)(k)(j\right)}=\frac{g_{\left(d)(k\right)}+g_{\left(d)(j\right)}}{2}$$

(A30) $${\bar{g}}_{\left(b)l(k)(j\right)}=\frac{g_{\left(b)l(k\right)}+g_{\left(b)l(j\right)}}{2}$$

Then, by taking the surface correction factors at the points $x_{\left(k\right)}$ and $x_{\left(j\right)}$ as the semi-axes of an ellipse, the value of the surface correction factor in any direction under simple loading conditions can be calculated as follows.

(A31) $$G_{\left(d)(k)(j\right)}={\left\{{\left[n_{1}/{\bar{g}}_{(d)(k)(j)1}\right]}^{2}+{\left[n_{2}/{\bar{g}}_{(d)(k)(j)2}\right]}^{2}\right\}}^{-1/2}$$

(A32) $$G_{\left(b)l(k)(j\right)}={\left\{{\left[n_{1}/{\bar{g}}_{(b)l(k)(j)1}\right]}^{2}+{\left[n_{2}/{\bar{g}}_{(b)l(k)(j)2}\right]}^{2}\right\}}^{-1/2}$$

in which $n_{1}$ and $n_{2}$ are obtained by the unit vector of the relative position vectors of $x_{\left(k\right)}$ and $x_{\left(j\right)}$.

(A33) $$n=\left[x_{\left(k\right)}-x_{\left(j\right)}\right]/\left|x_{\left(k\right)}-x_{\left(j\right)}\right|={\left\{n_{1},n_{2}\right\}}^{T}$$
